# The state of infant massage use in neonatal intensive care units

**DOI:** 10.1038/s41372-025-02488-7

**Published:** 2025-11-06

**Authors:** Dana B. McCarty, Polly Kellner, Natasha Mauger, Emma Bradford, Roberta Pineda

**Affiliations:** 1 Division of Physical Therapy, Department of Health Sciences, University of North Carolina at Chapel Hill School of Medicine, Chapel Hill, NC USA; 2 Chan Division of Occupational Science and Occupational Therapy, University of Southern California, Los Angeles, CA USA; 3 Department of Pediatrics, Keck School of Medicine, CA Los Angeles, USA; 4 Gehr Family Center for Health Systems Science and Innovation, University of Southern California, CA Los Angeles, USA

**Keywords:** Rehabilitation, Health occupations

## Abstract

**Objective:**

To characterize current infant massage practices in neonatal intensive care units (NICUs) and identify variability in approaches among neonatal therapists.

**Study design:**

A cross-sectional survey was distributed to NICU-based occupational therapists, physical therapists, and speech language pathologists. The survey inquired about massage use, training, protocols, techniques, and safety concerns. Descriptive statistics were used for analysis.

**Results:**

Among 101 respondents from 32 states, 90 (90%) used infant massage, with 64 (71%) considering it standard care. Infant massage was most often administered by occupational therapists (77, 76%), physical therapists (70, 69%), and parents (46, 46%). Despite high training rates (87, 97%), only 48 (53%) followed a specific protocol. Techniques, frequency, and use of emollients varied widely. Safety concerns included infant stress and physiological instability, though adverse events were rare.

**Conclusion:**

Despite widespread use of infant massage in NICUs, variability in findings underscores the need for standardization to ensure safe, effective delivery of massage.

## Introduction

From birth, preterm infants often require intensive, prolonged medical care in the neonatal intensive care unit (NICU). During this time, high risk of infant death and comorbidities creates a barrier to natural infant sensory-motor experiences and parent-infant bonding opportunities. Infant massage in preterm infants has been studied for more than 4 decades [1], with the first Cochrane Review published in 2000 [2]. More recently, infant massage has emerged as a promising intervention in NICUs as early as 32 weeks postmenstrual age (PMA) [3, 4] with benefits reported for both infants and parents [5, 6]. Numerous studies support its safety and efficacy when administered by skilled professionals or caregivers following appropriate training [5].

Research on infant massage has reported a range of infant physiological benefits such as increased oxygen saturation levels, enhanced vagal tone, improved gastric motility, and reduced pain, all of which contribute to shorter hospital stays and better post-discharge outcomes [7, 8]. Infant massage is also associated with improved anthropometric measures (e.g., weight, length, and head circumference) and improved oral feeding skills [3, 9]. Beyond these reported infant benefits, infant massage may also benefit parents who administer it to their infants by increasing opportunities for enhanced maternal-infant bonding and alleviating maternal mental health symptoms [6, 10].

There are specific protocols for infant massage, and some that were developed for different populations have been applied or adapted for infants in the NICU [9, 11–17]. However, the absence of standardized, evidence-based protocols for infant massage in the NICU limits its widespread adoption. Several reviews published between 2012 and 2025 [4, 5, 7, 18–20] have documented significant variability in infant massage protocols, including differences in duration, frequency, pressure, use of oils, and who administers the intervention. This heterogeneity in study design, intervention protocols, and implementation fidelity has significantly impeded the translation of infant massage research into standardized, evidence-based clinical practice that ensures both safety and efficacy.

The objectives of this study were to (1) characterize the current state of infant massage in clinical practice in NICUs across the United States from the perspective of neonatal therapists, and (2) identify variability in massage approaches across institutions and practitioners. Findings from this study may inform current research needs as well as the development of standardized protocols to optimize outcomes for hospitalized preterm infants.

## Subjects and methods

### Study design and participants

This cross-sectional study was approved by the Institutional Review Board at the University of Southern California (USC) with a waiver of written informed consent. All study procedures were conducted in accordance with relevant institutional guidelines and regulations and with the ethical principles outlined in the Declaration of Helsinki. Participants were provided with general information about study participation prior to survey initiation. Eligible participants included occupational therapists (OTs), physical therapists (PTs), and speech language pathologists (SLPs) currently practicing in the NICU. These disciplines were selected due to their central role in delivering sensory-based interventions, including infant massage, within neonatal care settings.

### Survey development

Survey prompts were developed by two neonatal therapy experts (DM, RP) following a comprehensive literature review and identification of gaps in existing knowledge [4]. The initial survey draft underwent iterative refinement by the full study team to improve clarity, reduce redundancy, and ensure relevance. Next, to enhance content validity, the survey was reviewed in three rounds by a total of 12 neonatal therapy (PT, OT, SLP) professionals. Each round included feedback from 3 to 5 therapists, with revisions incorporated after each round. By the third round, no new feedback was received, indicating consensus on survey content and structure.

The final survey was programmed in REDCap (Research Electronic Data Capture) [21], a secure, web-based application designed for data collection. Branching logic was used to tailor questions based on participants’ reported use of infant massage in their clinical practice.

### Recruitment and dissemination

Survey dissemination occurred between January and March 2025. A multi-pronged recruitment strategy was employed to maximize reach and diversity of respondents. Recruitment channels included professional social media platforms (e.g., Facebook groups, Instagram), email outreach to NICU therapy networks and colleagues, newsletters targeting neonatal therapists, and announcements at national neonatal therapy conferences. Participation was voluntary, and no incentives were provided. The survey link directed participants to the REDCap platform, where they could review the study information and proceed to the survey.

Survey responses were collected anonymously and stored securely within the REDCap system. Data were exported to StataMP16 for analysis [22]. Descriptive statistics were used to summarize participant responses. Open-ended responses were systematically reviewed and analyzed to identify recurring themes and patterns. Similar responses were combined and summarized to capture the essence of participants’ perspectives. Representative quotes were selected to illustrate common themes and provide insight into the range and depth of responses.

## Results

### Participant characteristics

A total of 101 responses were received from OTs, PTs, and SLPs currently practicing in the NICU. Survey responses represented 32 states in the U.S, as well as one respondent from each of the following: India, United Arab Emirates, Peru, and Guatemala. NICU levels spanned II-IV, with the majority working in Level III (59, 58%) and Level IV (37, 37%) units. Nine participants were from the same hospital as another participant (3 sets of 2 were from the same hospital, and one set of 3 participants were from the same hospital). The mean number of NICU beds reported by participants were 51.1 (31.4) beds (range 6-150), and therapists had a average of 12.3 (8.7) years (range 0.25-39) of NICU experience. Sixty four (63%) of respondents were Certified Neonatal Therapists (CNTs) [23] (Table 1).

**Table 1:** Participant and hospital characteristics.

	Mean (SD) or n^a^ (%)
NICU level	n = 101
Level II	5 (5%)
Level III	59 (58%)
Level IV	37 (37%)
Total number of beds	n = 100
	51.1 ± 31.4
Years of clinical experience in NICU	n = 100
	12.3 ± 8.7
Certified Neonatal Therapist (CNT)	n = 101
Yes	64 (63%)
No	37 (37%)
Use of specific program or sensory-based interventions in the NICU	n = 101
Yes	49 (49%)
No	52 (51%)
Name of sensory-based intervention or program used	n = 47
Supporting and Enhancing Neonatal Intensive Care Unit Sensory Experiences (SENSE)	45 (96%)
I-Rainbow	2 (4%)
The ladder approach	1 (2%)
Protocol developed locally	2 (4%)
Newborn Individualized Developmental Care and Assessment Program (NIDCAP)	1 (2%)
Massage used as a developmental intervention	n = 100
Yes	90 (90%)
No	10 (10%)
Infant massage is considered ‘standard of care’ developmental intervention	n = 90
Yes	64 (71%)
No	26 (29%)
Infant massage administered by	n = 101
Occupational therapist	77 (76%)
Physical therapist	70 (69%)
Parent	46 (46%)
Nurse	25 (25%)
Speech-language pathologist	16 (16%)
Developmental interventionist	3 (3%)
Child Life Specialist	1 (1%)
Music therapist	1 (1%)
Other	4 (4%)
Received formal or informal infant massage program	n = 90
Yes	87 (97%)
No	3 (3%)
Name of training program	n = 101
Neonatal touch and massage certification	69 (63%)
International Loving Touch Foundation	14 (13%)
Peer training	14 (13%)
International association of infant massage	7 (6%)
Field method	4 (4%)
Hospital to Home: Optimizing the Infant’s Environment (H-HOPE); Massage+30,10,5; Auditory, Tactile, Visual, and Vestibular stimulation (ATVV)	3 (3%)
Infant Massage USA, Certified Infant Massage Educator	1 (1%)
LiddleKidz or Certified Infant Massage Teacher (CIMT)	1 (1%)
Post-graduate fellowship training	1 (1%)
Shantala Foundation	1 (1%)
M-technique	1 (1%)
Use of a particular massage protocol	n = 90
Yes	48 (53%)
No	42 (47%)
Name of massage protocol used	n = 64
International Loving Touch Foundation	19 (21%)
Neonatal Touch and Massage	13 (14%)
Listening Touch	6 (7%)
Massage+30,10,5	6 (7%)
Infant Massage USA	5 (6%)
Eclectic mix of protocols	5 (6%)
International Association of Infant Massage	3 (3%)
Field Method	0 (0%)
M-technique	1 (1%)
Yakson	0 (0%)
Unstandardized	1 (1%)
None of the above	5 (6%)
Participant’s self-reported comfort administering infant massage (scale of 1–10 with 1 not comfortable to 10 completely comfortable	n = 98
	8.43 ± 2.25
(not comfortable) 1	1 (1%)
2	4 (4%)
3	1 (1%)
4	2 (2%)
5	4 (4%)
6	4 (4%)
7	4 (4%)
8	14 (14%)
9	17 (17%)
(completely comfortable) 10	47 (48%)

^a^n reflects number of respondents for each question.

### Use of infant massage in the NICU

Ninety (90%) of therapists respondents reported that infant massage was used as a developmental intervention in their NICU. Among these, 64 (71%) of therapists described infant massage as a standard of care practice. Therapists reported that infant massage was most commonly administered by OTs (77, 76%), PTs (70, 69%), and parents (46, 46%), with some units also involving nurses, developmental specialists, and massage therapists. Respondents who wrote in responses for “Other” indicated that trained volunteers, music therapists, nurses who underwent Neonatal Touch & Massage Certification (NTMC), and nurse practitioners also performed infant massage. Therapists reported a high level of comfort providing infant massage, with a mean comfort rating of 8.4 on a 10-point scale (1 = not comfortable, 10 = completely comfortable) (Table 1).

### Training and protocols

Among 90 respondents who reported using infant massage, 87 (97%) received formal or informal training. Fourteen (13%) were trained by peer therapists. Approximately 48 (53%) respondents indicated that their unit followed a specific massage protocol, while others reported using an eclectic mix of techniques. Although most participants received formal training through a specific infant massage program, use of the same training program’s protocol was not reported as frequently. For example, of the 64 respondents who reported use of a particular infant massage protocol, the most frequently cited training programs were the Neonatal Touch and Massage Certification (NTMC) (69, 68%) and International Loving Touch Foundation (ILTF) (14, 14%), yet only 13 (14%) and 19 (21%) respondents reported use of the NTMC and ILTF protocols, respectively. See Table 1.

### Massage techniques and environment

Table 2 provides reported characteristics for massage interventions. Massage was most often performed in an open crib (86, 96%), followed by the infant’s incubator (73, 81%) and in the lap (68, 76%). A kinesthetic, or movement, component was added after tactile stimulation in 48 (54%) of cases. In open responses, therapists described the kinesthetic components as joint compressions and/or approximations, range of motion, and facilitating movement towards midline (e.g., hand to mouth). Infant massage was integrated into multimodal interventions among 47 (53%) of respondents, often in combination with skin-to-skin care, music therapy, or other sensory-based interventions. R7rding pressure and speed, most respondents described using medium pressure (67, 74%) and slow strokes (77, 86%), with slow stroke durations descriptions ranging from 1-20 seconds, or 6 seconds on average. Only 2 respondents (2%) described infant pressure as “deep” and no therapists reported use of fast strokes. Respondents described a range of estimated infant massage frequencies from 1x/week (5, 6%) to more than 4x/day (1, 1%); however, most commonly reported infant massage frequencies were 3x/week (24, 29%) and 4x/week (17, 20%). Respondents estimated that parents administered infant massage approximately 37% ± 25% (range 0-100) of the time.

**Table 2:** Massage intervention characteristics.

	Mean (SD) or n^a^ (%)
Use of kinesthetic component following infant massage	n = 89
Yes	48 (54%)
No	41 (46%)
Type of kinesthetic component	n = 43
Facilitated movement/guided movement/stretching	14 (33%)
Joint compression or weight bearing	11 (26%)
Developmental positioning, pelvic tilts, or flexion movements	10 (23%)
Hand to mouth	4 (9%)
Myofascial techniques	4 (9%)
Midline orientation	3 (7%)
Rocking/vibration	3 (7%)
Grasp	2 (5%)
Dynamic or static touch	1 (2%)
Trigger reflexes largely into flexion	1 (2%)
Neurodevelopmental Treatment (NDT) intervention	1 (2%)
Massage is part of a multimodal intervention (e.g., kangaroo care, with music)	n = 89
Yes	47 (53%)
No	42 (47%)
Use of additional sensory inputs during massage	n = 43
Auditory (singing, speaking, music, humming)	24 (56%)
Skin-to-skin	19 (44%)
Vestibular/rocking/movement	5 (12%)
Gustatory/Olfactory (parent smell, tastes)	4 (9%)
Holding	2 (5%)
Swaddle bathing	2 (5%)
Visual	1 (2%)
Containment/proprioception	1 (2%)
Pacifier	1 (2%)
Proprioception	1 (2%)
Location of infant massage	n = 90
Open crib	86 (96%)
Inside the incubator	73 (81%)
On the caregiver/therapist’s lap	68 (76%)
During Kangaroo Care	21 (23%)
On the floor	17 (19%)
Other	5 (6%)
Describe the typical speed of the strokes during massage in strokes per second	n = 90
Slow	77 (86%)
Estimated strokes per second	Range 1-20; 6.3 (4.2)
Medium	13 (14%)
Estimated strokes per second	Range 1-9; 4.7 (2.5)
Fast	0 (0%)
Describe the typical amount of pressure used during massage	n = 90
Light	21 (23%)
Medium	67 (74%)
Deep	2 (2%)
Use of medium (e.g., emollient, oil) when providing massage	n = 101
Oil	45 (45%)
Lotion	23 (23%)
Aquaphor or Vaseline	18 (19%)
Massage over a swaddle or clothing with no emollient	23 (23%)
Massage over skin but without use of emollient	25 (25%)
Other or unstandardized	5 (5%)
Specific oil used	n = 45
Calendula oil	19 (42%)
Grapeseed oil	13 (29%)
Combination oil	5 (11%)
Coconut oil	4 (9%)
Sunflower oil	3 (7%)
Almond oil	1 (2%)
Safflower oil	1 (2%)
Jojoba oil	1 (2%)
Parent brings in oil	1 (2%)
Medium-chain-triglyceride oil	1 (2%)
Use of gloves during massage?	n = 90
Yes	32 (36%)
No	19 (21%)
Only in certain circumstances	39 (43%)
If yes, is there a unit policy for use of gloves during massage?	n = 32
Yes	31 (97%)
No	1 (3%)
Estimated massage frequency (times per week received)	n = 84
	4.41 ± 3.6
1 time per week	5 (6%)
2 times per week	11 (13%)
3 times per week	24 (29%)
4 times per week	17 (20%)
5 times per week	14 (17%)
6 times per week	1 (1%)
Daily	4 (5%)
1-2x Daily	7 (8%)
More than 4 times daily	1 (1%)
Estimated percentage of massage conducted by parents	n = 84
	(Range 0-99)
	37.6 ± 24.9

^a^n reflects number of responses for each question.

### Use of mediums and gloves

The use of emollients varied. Forty-eight (48%) respondents either massaged infants over clothing without an emollient or massaged directly on infant skin without using an emollient. Those who used a medium directly on infant skin during infant massage most commonly used oils (45, 45%). Open responses regarding the types of oil used for infant massage revealed a wide variety of preferences with calendula oil emerging as the most frequently reported (19, 42%). In open text responses, some respondents reported difficulty getting use of oil mediums approved in their NICUs due to fragile infant skin and unit policies. Gloves were always used during infant massage among 32 (36%) of respondents, and gloves were used only in certain circumstances when isolation, infection, or exposure precautions were in place among 39 (43%) of respondents. Open responses indicated that parents were encouraged to massage their infants without gloves to promote skin-to-skin contact and positive touch experiences.

### Goals of infant massage

Responses for indications and appropriate populations for massage are reported in Table 3. Respondents (n = 101) identified a wide range of goals for infant massage in the NICU. The most frequently endorsed infant-centered goals included providing positive sensory experiences (e.g., tactile, kinesthetic, proprioceptive input) (n = 88, 87%), promoting relaxation and/or sleep (86, 85%), and enhancing state regulation and/or facilitating calm alert states (85, 84%). Respondents also endorsed the following parent-centered goals: encouraging parent-infant interaction (84, 83%) and improving parent competence and responsiveness (84, 83%).

**Table 3:** Infant massage indications and appropriate populations.

	n^a^ (%)
Infant-Centered goals for massage	n = 101
Provide positive sensory experiences	88 (87%)
Promote sleep and relaxation	86 (85%)
Facilitate calm alert state/Enhance state regulation	85 (84%)
Reduce pain	85 (84%)
Improve infant tolerance to handling	84 (83%)
Improve respiratory function	84 (83%)
Improve skin texture	83 (82%)
Address withdrawal symptoms	82 (81%)
Improve feeding outcomes	79 (78%)
Weight gain	73 (72%)
Improve gastrointestinal issues	73 (72%)
Control edema	57 (56%)
Ease of diapering	57 (56%)
Improve tone and movement	53 (52%)
Improve bone density	45 (45%)
Improve bilirubin levels	34 (34%)
*Other (desensitization)	1 (1%)
Parent-centered goals for massage	n = 101
Facilitate parent-infant interaction	84 (83%)
Improving parent competence and responsiveness	84 (83%)
Increase parent engagement	74 (73%)
Infant conditions and clinical presentations that indicate massage	n = 101
Infants with challenges with state regulation	82 (81%)
Late preterm (33-36 weeks BGA)	78 (77%)
Hypertonia	76 (75%)
Neurological conditions (e.g., hypoxic ischemic encephalopathy)	62 (61%)
Hypotonia	61 (60%)
Infants with occupational therapy orders	60 (59%)
Infants with physical therapy orders	51 (50%)
Congenital anomalies	48 (48%)
Respiratory conditions	45 (45%)
Very low birth weight/very preterm ( < 32 weeks BGA)	31 (31%)
Infants with surgical conditions	29 (29%)
Cardiac conditions	28 (28%)
Extremely low birth weight/very preterm ( < 28 weeks BGA)	23 (23%)
Other	12 (12%)
PMA of infant when massage is typically initiated	n = 90
	Range 27–26 weeks PMA
	32.2 (1.4)
Massage is appropriate at the following PMAs	
23 weeks PMA	n = 88
Never	84 (95%)
Sometimes	4 (5%)
24 weeks PMA	n = 88
Never	82 (94%)
Sometimes	5 (6%)
25 weeks PMA	n = 87
Never	83 (95%)
Sometimes	5 (6%)
26 weeks PMA	n = 88
Never	78 (89%)
Sometimes	10 (11%)
27 weeks PMA	n = 87
Never	75 (86%)
Sometimes	12 (14%)
28 weeks PMA	n = 89
Never	61 (69%)
Sometimes	23 (26%)
About half of the time	5 (6%)
29 weeks PMA	n = 92
Never	54 (61%)
Sometimes	27 (30%)
About half of the time	8 (9%)
30 weeks PMA	n = 92
Never	41 (45%)
Sometimes	32 (35%)
About half of the time	12 (13%)
Frequently	7 (7%)
31 weeks PMA	n = 93
Never	35 (38%)
Sometimes	33 (35%)
About half of the time	14 (15%)
Frequently	11 (12%)
32 weeks PMA	n = 97
Never	5 (5%)
Sometimes	25 (25%)
About half of the time	26 (27%)
Frequently	29 (30%)
Always	10 (10%)
33 weeks PMA	n = 99
Never	2 (2%)
Sometimes	17 (17%)
About half of the time	23 (23%)
Frequently	46 (46%)
Always	11 (11%)
34 weeks PMA	n = 97
Sometimes	7 (7%)
About half of the time	11 (11%)
Frequently	61 (61%)
Always	18 (18%)
35 weeks PMA	n = 97
Sometimes	5 (5%)
About half of the time	6 (6%)
Frequently	63 (65%)
Always	23 (24%)
36 weeks PMA	n = 96
Sometimes	2 (2%)
About half of the time	6 (6%)
Frequently	59 (61%)
Always	29 (30%)
37 weeks PMA	n = 93
Sometimes	2 (2%)
About half of the time	3 (3%)
Frequently	59 (63%)
Always	29 (31%)
38 weeks PMA	n = 96
Sometimes	2 (2%)
About half of the time	1 (1%)
Frequently	61 (64%)
Always	32 (33%)
39 weeks PMA	n = 96
Sometimes	2 (2%)
About half of the time	1 (1%)
Frequently	61 (64%)
Always	32 (33%)
40 weeks PMA	n = 96
Sometimes	2 (2%)
About half of the time	1 (1%)
Frequently	58 (60%)
Always	35 (36%)
40+ weeks PMA	n = 96
Sometimes	2 (2%)
About half of the time	2 (2%)
Frequently	56 (58%)
Always	36 (38%)
Massage is appropriate in the following circumstances	
Within 72 hours for ELBW infant	n = 93
Never	87 (91%)
Sometimes	6 (6%)
Chest tube	n = 89
Never	27 (30%)
Sometimes	38 (43%)
About half of the time	8 (9%)
Frequently	14 (16%)
Always	2 (2%)
ECMO	n = 87
Never	59 (68%)
Sometimes	23 (26%)
About half of the time	3 (3%)
Frequently	2 (2%)
Endotracheal intubation	n = 92
Never	13 (14%)
Sometimes	40 (43%)
About half of the time	10 (11%)
Frequently	25 (27%)
Always	4 (4%)
Fragile bones	n = 92
Never	31 (34%)
Sometimes	41 (45%)
About half of the time	10 (11%)
Frequently	10 (11%)
Always	0 (0%)
During hypothermia	n = 91
Never	72 (79%)
Sometimes	13 (14%)
About half of the time	4 (4%)
Frequently	2 (2%)
Non-invasive ventilation	n = 95
Never	10 (11%)
Sometimes	27 (28%)
About half of the time	16 (17%)
Frequently	33 (34%)
Always	9 (10%)
Neonatal abstinence syndrome or neonatal opioid withdrawal syndrome	n = 97
Never	2 (9%)
Sometimes	3 (3%)
About half of the time	4 (4%)
Frequently	52 (54%)
Always	36 (37%)
Open wounds	n = 92
Never	57 (62%)
Sometimes	31 (33%)
About half of the time	2 (2%)
Frequently	1 (1%)
Sepsis	n = 93
Never	75 (81%)
Sometimes	11 (12%)
About half of the time	3 (3%)
Frequently	4 (4%)
Infant body parts avoided during massage	n = 65
Fragile bone, limb at risk of deep vein thrombosis; skin infection, rash, open wound	16 (25%)
Abdomen/belly	12 (18%)
Genitals	7 (11%)
Limbs with intravenous or catheter lines (PIV, PICC)	8 (12%)
Anterior chest	6 (9%)
Belly button/umbilical area	4 (6%)
Head	4 (6%)
Face	4 (6%)
Spine	2 (3%)
A painful limb	1 (2%)
Safety issues observed during massage	n = 101
Behavioral motor stress signs	62 (61%)
Infant physiological instability	36 (36%)
Respiratory changes	14 (14%)
Temperature instability	13 (13%)
Lines concerns (dislodgment, pulling)	9 (9%)
Allergic response to medium	6 (6%)
Skin safety	4 (4%)
Other (movement of nasal prongs)	1 (1%)

^a^n reflects number of responses for each question.

### Infant eligibility and timing

The average PMA reported for initiation of infant massage was 32.2 (1.4) weeks (range 27–36). Infant conditions and clinical presentations that indicated massage as part of the therapy plan of care were most commonly state regulation dysfunction (82, 81%), and infants born at late preterm gestation (78, 77%). The most endorsed conditions for initiating infant massage “frequently” or “always” included Neonatal Opioid Withdrawal Syndrome (NOWS) (87, 90%). Open text responses to “Other” populations (12, 12%) discussed use of infant massage as part of an early mobilization protocol for infants undergoing extracorporeal membrane oxygenation (ECMO) and continuous renal replacement therapy (CRRT). In contrast, the majority of respondents identified ECMO (59, 66%) as one of several clinical conditions under which infant massage is never initiated.

Few respondents endorsed initiating massage before 32 weeks PMA, however, 4 (4%) of respondents reported that massage was “sometimes” appropriate as early as 23–24 weeks PMA. Open responses mentioned considerations of birth gestational age (BGA), medical readiness, and physiological stability to determine the appropriateness of massage, with some presenting guidelines discouraging massage for infants <1500 g or those with active infections, sepsis, or unhealed surgical sites.

### Safety concerns

When asked about safety issues observed during massage, respondents (n = 101) most frequently reported infant stress (62, 61%) and physiological instability (e.g., changes in heart rate or oxygen saturation) (36, 36%), with fewer respondents concerned about allergic reactions to massage mediums (6, 6%) or skin safety (4, 4%). Open-ended responses about safety concerns discussed the importance of monitoring stress cues, ensuring thermoregulation, and adjusting interventions based on infant tolerance or behavior. Additional concerns were noted about potential safety issues with insufficient caregiver training and pressure inconsistencies.

### Narrative description of infant massage

Finally, respondents were invited to describe infant massage practices in their unit using the following free-text prompt: *“Describe the massage you do in your unit. (What is the environmental set-up, position of the infant, where are your hands, where do you start, how many strokes, what body parts are included, how much pressure is applied?)”* Of the 101 individuals surveyed, 78 (77%) provided detailed responses. As noted in previous multiple-choice responses, open-ended responses revealed considerable variability in infant massage practices across neonatal units; however, common themes emerging in environmental set-up, infant positioning, techniques, and responsiveness to infant cues are described below.

Regarding environmental setup, infant massage was typically performed in low-stimulation environments, including incubators, open cribs, or on the therapist’s or parent’s lap. Dim lighting, warm blankets, and quiet surroundings were frequently used to promote infant comfort. Massage sessions were often integrated into care routines, such as before or after feeding or diapering. Regarding positioning, infants were usually positioned in prone, side-lying, or supine positions depending on their medical stability and tolerance. Respondents described use of semi-swaddling to contain the infant, with only the body part being massaged exposed. Sessions typically began with static touch and progressed to dynamic strokes of commonly massaged areas: back, extremities, and face. Abdominal massage, such as the “I Love You” technique, was used to support gastrointestinal function. Some therapists reported use of scar massage or manual edema mobilization when clinically indicated. Number of strokes per body part ranged from 3 to 10, though many respondents emphasized that stroke count was determined by infant cues rather than a fixed protocol. Pressure was described as light to moderate, often guided by visual cues such as slight skin blanching or wrinkling. In general, respondents reported that infant massage was paused or discontinued if the infant demonstrated stress cues.

## Discussion

This study offers an in-depth description of infant massage practices in NICUs, revealing its widespread use, substantial variability in technique, and shared safety concerns. Survey responses demonstrate that neonatal therapists not only recognize the potential benefits of infant massage but also advocate for its integration into clinical practice. Additionally, the data suggest that these clinicians are well-informed regarding the evidence base for infant massage, supporting its indications and therapeutic benefits.

While over 70% of respondents reported that massage was considered standard of care practice in their NICU, a central finding of this study was the significant variability in infant massage techniques and protocols described—an observation that mirrors the heterogeneity documented in prior reviews [5, 7, 18, 20]. Across the reviewed literature, infant massage protocols described share several core characteristics, including the use of tactile and kinesthetic stimulation, moderate pressure techniques, and structured session durations typically ranging from 10 to 20 minutes. However, reported protocols differ in stroke speed, weekly frequency, and use of emollients, with no universally accepted standards [5, 7, 18, 20]. A 2015 meta-analysis [20] and more recent reviews by Alvarez et al. [7] and Pados et al. [5] highlight similar inconsistencies, particularly in how “moderate pressure” is defined and applied, despite its use in infant massage being associated with improved weight gain and physiological outcomes.

Estimated frequency of infant massage in the NICU varied considerably in our study, from 1x/week to 4x/day, with 3-4x/week most commonly reported. These variables and mostly low frequencies of massage perhaps stand in the greatest contrast to one of the most researched infant massage protocols, the Field Method [16, 17], which employs infant massage 3 times per day. The Field Method has been associated with positive outcomes [16, 17, 24], but it is unclear if infants receive the same degree of positive benefits when massage is delivered only 2–3x/week as reported by our survey respondents. Two small clinical trials published within the last 2 years examined two different infant massage protocols employed daily over a 2-week period in preterm infants >30 weeks PMA with opposing results. A study by Weerakul et al. [25] demonstrated increased weight gain and reduced hospital stays in the massage group—consistent with earlier studies employing infant massage at a frequency of 3 times daily. In contrast, a trial by Alinejad-Naeini et al., which evaluated the M-Technique massage protocol [9], did not find significant differences in infant weight gain between intervention and control groups; however, the intervention group demonstrated more favorable behavioral response scores than the control group—findings that may have important implications for parent-infant interaction [26].

Use of ILTF and NTMC protocols was most frequently reported, yet, despite even higher reported rates of formal training in these protocols, many therapists appear to opt for eclectic approaches that draw from multiple sources or personal experience. This variability may be attributed to the fact that, although ILTF and NTMC protocols are accessible to therapists who have completed training programs, continued access to parent education materials, program resources, and updated protocols often requires re-certification or annual membership fees. These additional requirements may discourage therapists or institutions from committing to a specific program or protocol [11, 12].

Survey responses indicated that use of emollients or mediums varied considerably. When mediums were not used, open-text responses revealed that it was typically because unit policies prohibited emollients in the NICU due to concerns about the fragility of preterm infants’ skin. Topical emollients are more commonly used in low- to middle-income countries due to cultural norms that support oil massage for newborns and because they offer a low-cost, accessible way to reduce infection risk and support skin barrier function [27]. Evidence from clinical trials in these regions has demonstrated benefits such as improved skin integrity, enhanced growth, and reduced neonatal mortality, yet, according to the 2023 WHO recommendations, topical emollient use is conditionally recommended due to “low certainty” evidence on type, dose, timing, and duration [28]. The report notes that sunflower or coconut oil may be appropriate with gentle application to protect skin integrity and that use of emollients should be guided by clinical judgment and in collaboration with parents [28].

Although emollient therapy is used globally to support skin integrity and reduce infection rates, in the US, neonatal therapists primarily use a medium during infant massage to promote comfort and relaxation. An early study by Field et al. [29] found that typically developing 1-month old infants massaged with oil showed fewer limb movements, fewer stress behaviors, less gaze aversion, and greater physiological regulation, including lower salivary cortisol levels during massage than those massaged without oil. A follow-up study by Field et al. in 2016 [30] found that full-term, healthy newborns who received daily massage with lotion slept longer and experienced fewer night wakings over a one-month period compared to those massaged without lotion. Therefore, more research is needed to identify the safest emollient for use on preterm infant skin in the context of infant massage application.

Respondents to our survey felt that infant massage’s application should be approached with caution, particularly for infants under 32 weeks PMA, those undergoing ECMO, or those with chest tubes, sepsis, or open wounds. These findings reflect high levels of clinical decision making and align with prior studies that have emphasized the need for careful assessment of infant medical stability before initiating massage [31]. Inappropriate infant PMA and medical conditions described in our results are aligned with recent clinical trials of preterm infant massage which excluded infants <28 weeks PMA and <30 weeks PMA, infants with congenital anomalies (e.g., congenital heart disease, congestive heart failure, lung or airway malformations), requiring endotracheal intubation or chest tubes, exhibiting unstable vital signs (including persistent bradycardia or tachycardia), diagnosed with convulsions, septic shock, or grade III/IV intracranial hemorrhage, or experiencing severe respiratory distress. While some therapists reported performing infant massage on infants with high-acuity conditions, such as those receiving ECMO, these practices were not widely endorsed and highlight the need for clearer contraindication guidelines.

Safety concerns were recognized by most respondents, with 61% having observed infant behavioral stress signs and 36% observing physiological instability during infant massage. These concerns were generally managed through responsive clinical decision making, such as adjusting pressure or speed, pausing, or discontinuing massage. Systematic reviews have identified adverse effects such as changes in heart rate, oxygen saturation, and skin integrity when infant massage is applied without appropriate clinical oversight [31], but overall, conclude that adverse events during infant massage are extremely rare [7]. However, safety issues or adverse events are not consistently reported in published research.

Although strategies for managing adverse events in the NICU are well-documented in the literature [32, 33], most studies focus on medication-related safety concerns, and no existing reviews specifically address adverse events associated with infant massage. Gao et al. investigated the effectiveness and safety of combining sucrose with massage, music, non-nutritive sucking, and gentle human touch to manage repeated procedural pain in preterm infants. As a secondary outcome, they monitored adverse events such as vomiting, abdominal distension, coughing, choking, oral infection, sustained tachycardia, bradycardia, tachypnea, dyspnea, and hyperglycemia before, during, and after procedures. Among the 71 infants studied, vomiting was the only adverse event reported, occurring in one infant from the routine care group and one from the intervention group [34]. Given the limited reporting of adverse events in systematic reviews and our survey findings, neonatal therapists appear aware of potential risks but generally view the benefits of infant massage as outweighing those risks. Authors of a systematic review published in 2017 recognized the need for adaptive, cue-based approaches that account for infant medical status and individual tolerance during infant massage [7], which aligns with how neonatal therapists approach infant massage based on open-text responses in our survey.

### Limitations

This study has limitations. The survey format and means of recruitment may have led to underrepresentation of certain groups, particularly those in Level II NICUs or units without dedicated neonatal therapists. Most respondents were therapists who regularly perform infant massage and who may have a positive bias toward its benefits, potentially skewing the data. Additionally, while other disciplines involved in infant massage were mentioned, this survey included only neonatal therapists so lacks representation from non-therapy providers who may also administer massage. Duplicate responses from the same hospital were identified and addressed, but this may still influence the generalizability of findings. Further, despite our multi-pronged recruitment approach to increase our reach, we acknowledge that our response rate of 101 therapist respondents represents only a small fraction (<1%) of the estimated 4232 neonatal therapists in the US [35]. Additionally, we acknowledge that because respondents represented various areas of the US and outside of the US, there may be cultural differences in massage characteristics reported and this is an important area to consider for future research.

## Conclusion

Infant massage is a widely used and valued intervention in the NICU, yet its implementation is marked by considerable variability and clinical caution. Therapists adapt their approaches based on infant cues and medical status, suggesting that flexibility is both common and necessary in this complex environment. While safety concerns are not uncommon, they appear to be managed with thoughtful clinical judgment. Based on these results, the majority of neonatal therapists are aware of the indications and benefits of infant massage based on available evidence. These findings highlight the need not only for clearer definitions in infant massage techniques and more transparent and reproducible infant massage protocols in research, but also for standardized, evidence-based protocols and training to ensure safe and effective infant massage practices in neonatal care.

## Data Availability

The datasets generated and/or analyzed during the current study are available from the corresponding author on reasonable request.
